# Probing Slip Differential Heat of Magnetorheological Fluids Subjected to Shear Mode Operation and Its Effect on the Structure

**DOI:** 10.3390/ma12111860

**Published:** 2019-06-08

**Authors:** Song Chen, Jing Yang

**Affiliations:** College of Mechanical Engineering, Chongqing University of Technology, Chongqing 400054, China; songchen1133@163.com

**Keywords:** magnetorheological fluids, slip differential heat, shear mode, temperature, thermal structure analysis, slip power

## Abstract

The paper probes slip differential heat of magnetorheological fluids (MRFs) subjected to shear mode operation and its effect on the structure. To begin, we present a novel model for measurement of slip differential heat to describe temperature rise of MRFs mainly caused by friction between magnetorheological particles. It includes two stages: (1) The micro-macro analysis of slip differential heat of MRFs including force, movement and heat between neighboring particles based on magnetic dipole and Hertzian contact theories, and (2) the further application to two basic disc-type and cylinder-type magnetorheological clutches combined with finite element simulations involving electromagnetic field and thermal analysis. The model takes into account the effect of each of the main influencing factors, such as the input current of excitation coil, the rotational speed difference of the clutches, the size and volume fraction of particles, the saturation magnetization of particles, and the structural size of the clutches, etc., on the slip differential heat of MRFs. Then the thermal structure analysis of MRFs comprising thermal deformation and equivalent thermal stress is carried out. Moreover, the effect of typical governing parameters on the slip power of MRFs and the influence of slip differential heat on the structure of MRFs are investigated individually. We show that such a model is effective in reflecting the temperature-slip time relation of MRFs. It is shown that the input current and the rotational speed difference have great effect on the slip power, and the slip differential heat has a certain influence on the micro-structure of MRFs.

## 1. Introduction

Magnetorheological fluids (MRFs) are a type of controllable and designable smart material mainly composed of micron-sized ferromagnetic particles, a non-ferromagnetic carrier and a stabilizer. They can translate reversibly from a Newtonian fluid into a semi-solid with a tunable shear stress in milliseconds upon the application of an external magnetic field and demonstrate dramatic changes in their macroscopic rheological characteristics, which has made MRFs attract considerable interest for application in many engineering devices, such as engine mounts, clutches, brakes, and dampers [1,2,3,4,5]. When MRFs serve as a transmission medium in a magnetorheological clutch (MRC) or magnetorheological brake subjected to the shear mode operation, the fluid temperature obviously increases due to the slip differential heat of MRFs [6]. The temperature rise causes the work performance of MRFs to decrease or even fail since the carrier is a temperature-dependent material [7]. Besides, it has a certain influence on the structure because the size of MRFs is very small and the magnetorheological particles are micron-sized.

In recent years, great efforts have been made to investigate the slip differential heat of MRFs subjected to the shear mode operation. Kavlicoglu et al. [8] attributed the heating sources of an MRC to electrical power input and viscous slippage between clutch plates, and measured the temperature rise of the casing surface and side caps of the MRC caused by these heating sources through experiments. Furthermore, they performed a theoretical analysis to obtain the temperature of the MRC as a function of time through a lumped system approach [9]. However, there is a large difference between the obtained temperature of the lumped system analysis and the actual result; and the temperature distribution in the MRC, especially MRFs, is hard to accurately determine. These shortcomings make this theoretical method difficult to be applied to more problems. Moreover, Parker et al. [10] thought the slip differential heat is caused by the friction between the MRF walls and disk surfaces. They conducted the magneto static simulations and supplied the magnetic field distribution to a computational fluid dynamics simulation to obtain the temperature distribution within the magnetorheological brake [10]. And then Karakoc et al. [11] carried out a follow-on study to the work conducted by Parker et al. [10]. They further divided the slip differential heat sources into two cases: (1) Heat generated when there is no applied magnetic field, and (2) heat generated when a magnetic field is present [11]. The results show that the temperature increase in the first case is pretty small, so the effect is negligible [11]. These analyses do not take into account the material components and working mechanism of MRFs. In addition, Wang et al. [6,12,13,14,15] attributed the slip differential heat of MRFs to the frictional interaction among the magnetorheological particles [6], but they did not further formulate a theoretical scheme for it. They investigated the effect of many main factors, such as the slip power [12], input current [13] and cooling conditions [13,14,15], on the temperature variation of MRFs. Besides, Song et al. [16] investigated the slip differential heat and heat dissipation of the magnetorheological brake under different working gaps. The results of the research indicated that the temperature caused by the slip differential heat is higher under the smaller working gap contrast to the larger gap [16]. Wang et al. [17] studied the effect of the slip differential heat and heat dissipation on the torque of a high-torque squeezing magnetorheological brake and obtained the relationship between the torque and temperature by conducting a temperature–torque performance experiment [17]. However, these studies [8,9,10,11,12,13,14,15,16,17] do not analyze the slip differential heat of MRFs based on micro-structures and micro-mechanics since macro phenomenon is determined by micro nature. Meanwhile, there is no study on the effect of the slip differential heat on the structure of MRFs.

A previous study by the present authors has studied in detail the principal of slip differential heat generation of MRFs in each stage from the micro-scale, and a series of assumptions for the theoretical analysis have been made [18]. In the present paper, the slip differential heat of MRFs subjected to shear mode operation and its effect on the structure are probed. To begin, we analyze the magnetic force between neighboring particles, the slip differential heat flow of particle chains, and the slip power of MRFs throughout the shear deformation according to the chaining and shear mechanism of MRFs. For the application of MRFs to clutches subjected to the shear operation, we describe two basic disc-type and cylinder-type magnetorheological clutches combined with finite element simulations involving an electromagnetic field and transient thermal analysis. Then we carry out the thermal structure analysis of MRFs comprising thermal deformation and equivalent thermal stress. Through the comparison with the experimental measurements of the temperature variations of MRFs, the effectiveness of the proposed model is validated. In addition, we investigate the effect of typical governing parameters, such as the input current of excitation coil and the rotational speed difference of the clutches, on the slip power of MRFs and the influence of slip differential heat on the structure of MRFs.

## 2. Micro-Macro Model of Slip Differential Heat of MRFs

**Figure 1:**
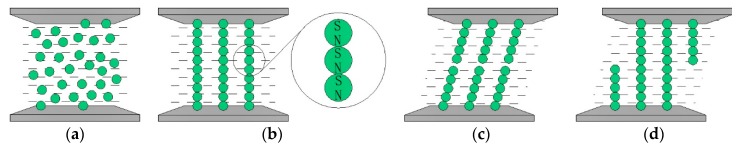
Shear motion of particles: (**a**) Initial state; (**b**) Chaining; (**c**) Stretching; (**d**) Reformation.

It is known that ferromagnetic particles disperse randomly in a non-magnetic carrier liquid without the application of an external magnetic field, see Figure 1a. Once a magnetic field is applied, the particles aggregate instantly into chains aligning in orientation to the magnetic field [19], see Figure 1b. It is worth noting that the magnetic induction intensity of the magnetized particle is affected by the induced magnetic fields produced by other particles, but Reference [18] showed that the induced magnetic field produced by the neighboring particle is a much more significant energy source. Also, the distance between chains is sufficiently large so that the interaction between the particles in different chains is negligible [20]. Besides, it is assumed that all the particles are made of the same material and are of spheres with identical radius. Thus, the effect between two neighboring particles in a single chain is only considered in this study. As shown in Figure 1b, each magnetized particle has two magnetic poles similar to N and S poles and there are a pair of attractive magnetic forces between two neighboring particles. Thus, the magnetic force between two neighboring particles (particle *i* and *j* = *i* + *1*) is expressed as [21]

(1) $$F_{mi}=\underset{j\neq i}{\sum }\left[\frac{3\mu_{0}}{4\pi r_{ij}^{5}}\left(m_{i}\cdot m_{j}-5m_{ir}m_{jr}\right)r_{ij}+\frac{3\mu_{0}}{4\pi r_{ij}^{4}}\left(m_{ir}m_{j}+m_{jr}m_{i}\right)\right]$$

where *μ*_0_ is the permeability in free space, *r_ij_* is the distance between the centers of particle *i* and particle *j*, **m***_i_* and **m***_j_* are the magnetic moments of particle *i* and particle *j*, **r***_ij_* is the vector designating the relative position from particle *j* to that of particle *i*, and *m_ir_* and *m_jr_* are the components of magnetic moment **m***_i_* and **m***_j_* in the direction of **r***_i_*.

In addition to the magnetic force, the particle is subjected to other forces including electro-repulsive force, viscous resistant force, van der Waals force, brown force, gravity and buoyancy, etc. [22]. However, the research demonstrated that the van der Waals force, brown force, gravity and buoyancy are much smaller than the magnetic force, electro-repulsive force and viscous resistant force so that the present paper only considers the magnetic force, electro-repulsive force and viscous-resistant force [22]. The electro-repulsive force and viscous-resistant force are respectively given by [23,24]

(2) $$F_{vi}=-6\pi R\eta u$$

(3) $$F_{ri}=\underset{j\neq i}{\sum }\frac{3\mu_{0}m_{i}{}^{2}}{2\pi r_{ij}^{4}}\exp\left[-\beta \left(\frac{r_{ij}}{2R}-1\right)\right]r_{ij}$$

where *R* is the radius of the particle, *η* is the viscosity of the carrier, and ***u*** is the velocity of the particle.

Based on the magnetic dipole theory, the magnetic moment of each magnetized particle is determined as

(4) $$m=VM=\frac{4}{3}\pi R^{3}\chi H$$

where *V* is the volume of the particle, **M** is the intensity of magnetization, *R* is the radius of the particle, *χ* is the susceptibility of the particle, and **H** is the magnetic field.

The exact determination of the susceptibility *χ* is complicated since the magnetization of the particle is nonlinear and related to many influencing factors. However, under consideration of magnetization saturation, it can be approximately described by the Frohlisch-Kennelly equation [25].

(5) $$\chi =\frac{\chi_{0}M_{s}}{\chi_{0}H+M_{s}}$$

where *H* is the magnetic field intensity, *χ*_0_ is the initial susceptibility when *H* tends to zero, and *M_S_* is the saturation intensity of magnetization.

Alternatively, assuming *θ_ij_* is the angle between the **r***_ij_* orientation ${\vec{r}}_{ij}$ and the orientation of the magnetic field $\vec{y}$ and substituting Equations (4) and (5) into Equations (1)–(3) yields

(6) $$F_{mi}\left(\theta_{ij}\right)=\underset{j\neq i}{\sum }\frac{\pi \mu_{0}\chi_{0}^{2}M_{s}^{2}H^{2}R^{2}}{12{\left(\chi_{0}H+M_{s}\right)}^{2}}\left[\left(1-5cos^{2}\theta_{ij}\right){\vec{r}}_{ij}+2cos\theta_{ij}\vec{y}\right]$$

(7) $$F_{vi}=-6\pi R\eta v_{\tau }\vec{x}$$

(8) $$F_{ri}=-\frac{\mu_{0}\pi R^{2}\chi_{0}^{2}M_{s}^{2}H^{2}}{6{\left(\chi_{0}H+M_{s}\right)}^{2}}{\vec{r}}_{ij}$$

where *F_mi_*(*θ_ij_*), *F_υi_* and *F_ri_* are respectively the magnitude of the magnetic force, electro-repulsive force and viscous resistant force, respectively; *υ_τ_* is the relative velocity between the upper and lower walls, and $\vec{x}$ is parallel to the orientation of the magnetic field.

For two neighboring particles (particle *i* and *j* = *i* + *1*), the radial components of the mutual forces *F_ij_* and *F_ji_* are derived as

(9) $$F_{ij}=\frac{\pi \mu_{0}\chi_{0}^{2}M_{s}^{2}H^{2}R^{2}}{12{\left(\chi_{0}H+M_{s}\right)}^{2}}\left(1-3cos^{2}\theta_{ij}\right)+6\pi R\eta v_{\tau }sin\theta_{ij}-\frac{\mu_{0}\pi R^{2}\chi_{0}^{2}M_{s}^{2}H^{2}}{6{\left(\chi_{0}H+M_{s}\right)}^{2}}$$

(10) $$F_{ij}=-\frac{\pi \mu_{0}\chi_{0}^{2}M_{s}^{2}H^{2}R^{2}}{12{\left(\chi_{0}H+M_{s}\right)}^{2}}\left(1-3cos^{2}\theta_{ij}\right)+6\pi R\eta v_{\tau }sin\theta_{ij}+\frac{\mu_{0}\pi R^{2}\chi_{0}^{2}M_{s}^{2}H^{2}}{6{\left(\chi_{0}H+M_{s}\right)}^{2}}$$

According to Hertzian contact theory, the radius and the maximum contact stress of the contact surface between two neighboring particles are determined as

(11) $$a_{ij}={\left[\frac{3R\left(1-\mu^{2}\right)\left(F_{ij}-F_{ji}\right)}{4E}\right]}^{\frac{1}{3}}$$

(12) $$q_{ij}={\left[\frac{6E^{2}\left(F_{ij}-F_{ji}\right)}{\pi^{3}R^{2}{\left(1-\mu^{2}\right)}^{2}}\right]}^{\frac{1}{3}}$$

where *E* is the elastic modulus of the particle, and *μ* is the Poisson ratio of the particle.

**Figure 2:**
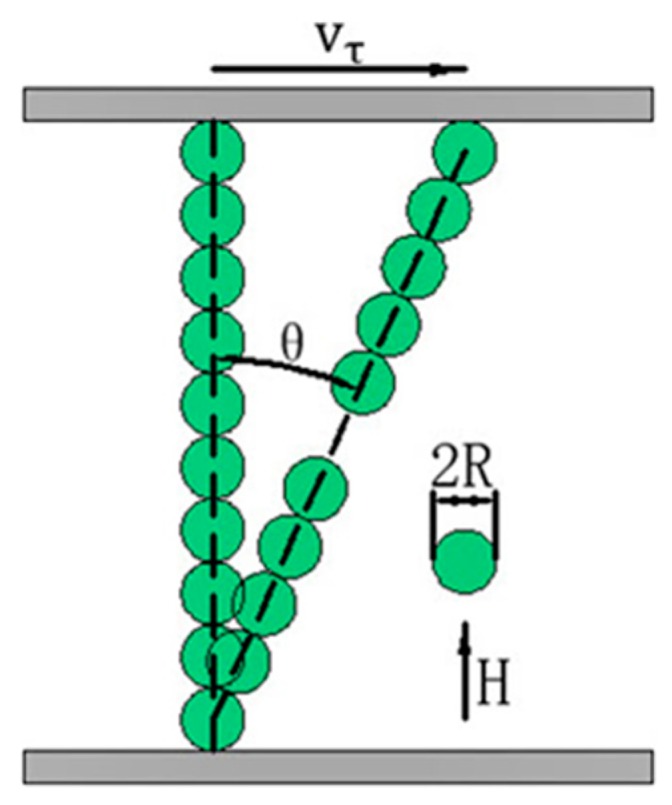
Shear deformation of a single chain.

There is a relative motion between two neighboring particles when MRFs are subjected to a shear deformation, see Figure 1c. In addition, the work of Furst et al. [26] demonstrates that the single chain stretches and breaks between any two neighboring particles during the deformation process as shown in Figure 2 which illustrates the shear deformation of a single chain. For the two stretched neighboring particles in a single chain, there is no friction between them due to no contact. However, for the other unstretched neighboring particles in a single chain they rub against each other, which causes the temperature rise of MRFs. Thus, the slip differential heat flow between two neighboring particles is derived as

(13) $$J_{ij}\left(\theta_{ij}\right)=\frac{2\mu_{k}}{3}q_{ij}\times \pi a_{ij}^{2}\times v_{ij}=\frac{2\mu_{k}v_{\tau }}{N-1}\left[\frac{\pi \mu_{0}\chi_{0}^{2}M_{s}^{2}H^{2}R^{2}}{12{\left(\chi_{0}H+M_{s}\right)}^{2}}\left(1-3cos^{2}\theta_{ij}\right)-\frac{\mu_{0}\pi R^{2}\chi_{0}^{2}M_{s}^{2}H^{2}}{6{\left(\chi_{0}H+M_{s}\right)}^{2}}\right]$$

where *μ_k_* = 0.2 is the friction coefficient of the particle surface, *υ_ij_* the relative velocity between two neighboring particles, and *N* is the number of particles in a single chain.

During the shear deformation of a single chain, the angle *θ_ij_* continually increases with the shear strain. When it reaches the maximum *θ*_0_ [20], the chain will break and re-form and a similar process will be repeated, see Figure 1d. The time of the break and re-formation is sufficiently short so that the slip differential heat flow during these processes can be negligible. Thus, the average slip differential heat flow of a single chain is given by

(14) $$\bar{J_{c}}=\frac{1}{\theta_{0}}\overset{N-1}{\underset{i=1}{\sum }}\int_{0}^{\theta_{0}}J_{ij}\left(\theta_{ij}\right)d\theta_{ij}=\frac{\pi \mu_{k}\mu_{0}R^{2}\chi_{0}^{2}M_{s}^{2}H^{2}v_{\tau }}{24{\left(\chi_{0}H+M_{s}\right)}^{2}}\left(7-\frac{3sin2\theta_{0}}{\theta_{0}}\right)$$

For MRFs with the volume fraction of particles *ψ*, suppose the number of particles in a single chain keeps constant during the shear deformation and suppose all the chains possess the same number of particles [27]. The number of chains per unit area is estimated with

(15) $$Z=\frac{\psi Sh}{SN\left(4\pi R^{3}/3\right)}=\frac{3\psi }{2\pi R^{2}}$$

where *S* is the area of MRFs perpendicular to the orientation of the magnetic field, and *h* = 2*RN* is the spacing between the upper and lower walls.

Therefore, the slip power of MRFs is derived as

(16) $$P_{m}=SZ\bar{J_{c}}=\frac{\mu_{k}\mu_{0}\chi_{0}^{2}M_{s}^{2}H^{2}v_{\tau }S\psi }{16{\left(\chi_{0}H+M_{s}\right)}^{2}}\left(7-\frac{3sin2\theta_{0}}{\theta_{0}}\right)$$

## 3. Model of Slip Differential Heat of Magnetorheological Clutches

### 3.1. Principle of Two Basic Disc-Type and Cylinder-Type Magnetorheological Clutches

**Figure 3:**
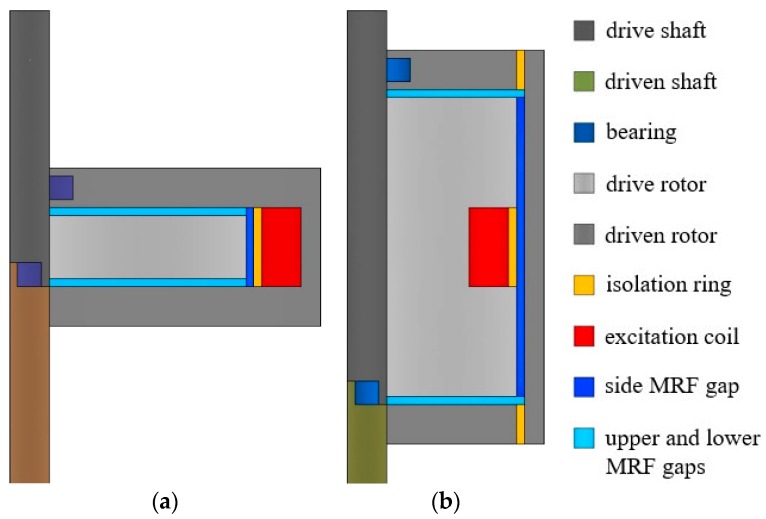
Half-section views of the clutches: (**a**) Disc-type MRC; (**b**) Cylinder-type MRC.

It is well known that MRFs can be applied to the disc-type and cylinder-type magnetorheological clutches subjected to shear operation [28]. Two basic disc-type and cylinder-type magnetorheological clutches are presented as shown in Figure 3, which illustrates half-section views of the clutches in detail. It can be seen from the figure that the drive rotors and the driven rotors are respectively connected to the drive shafts and the driven shafts, and there are MRF gaps filled with MRFs between the drive rotors and driven rotors. When the excitation coil is not energized, MRFs exhibit a free-flow state and the drive rotor and the driven rotor are disengaged. However, the shear stress of MRFs is greatly enhanced and the drive rotor and driven rotor are engaged once the excitation coil is energized. Moreover, the isolation rings are used to guide the magnetic flux and reduce the magnetic flux leakage. It is known that for the disc-type MRC, see Figure 3a, only MRFs in two upper and lower MRF gaps can be used to transmit the torque on one hand, and on the other hand, it is contrary to that for the cylinder-type MRC, see Figure 3b. That is because the distributions of excitation coils and the magnetic circuits are different in the disc-type MRC and the cylinder-type MRC. Therefore, the volumes of the working MRFs of the disc-type MRC and the cylinder-type MRC were designed to be approximately the same so that we can better compare their characteristics of slip differential heat. Based on the realistic situation, some characteristic design parameters of the clutches used in this study are shown in Table 1.

### 3.2. Electromagnetic Field Analysis

**Figure 4:**
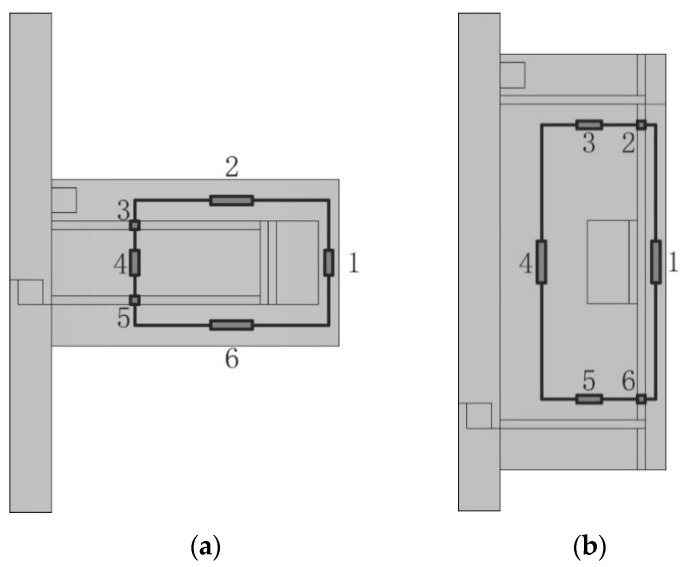
Magnetic circuits of the clutches: (**a**) Disc-type MRC; (**b**) Cylinder-type MRC.

It can be seen from Equation (16) that the slip power *P_m_* is related to the magnetic field intensity *H* determined by the input current of the excitation coil *I*. To obtain the relationship *H*(*I*) between the magnetic field intensity and the input current, magnetic flux is inserted between them for transition. On one hand, the magnetic flux is in detail determined by the magnetic field intensity in Reference [11], on the other hand, the magnetic flux is in detail given by the input current in Reference [12] combined with Figure 4 which illustrates a closed magnetic flux path.

**Figure 5:**
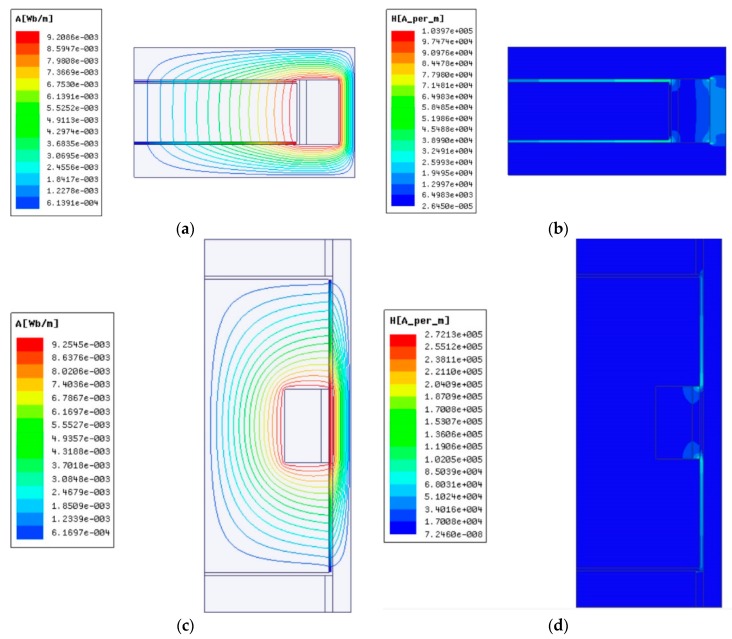
Results of electromagnetic field analysis: (**a**) Magnetic flux distribution of the disc-type MRC; (**b**) Magnetic field intensity of the disc-type MRC; (**c**) Magnetic flux distribution of the cylinder-type MRC; (**d**) Magnetic field intensity of the cylinder-type MRC.

In order to obtain the exact relationship between the input current and the corresponding magnetic field intensity, the electromagnetic field simulations of the clutches were carried out by commercial finite element software ANSYS-Maxwell® 16.0 [29]. The simulations can be simplified to a two-dimensional plane problem since the model, the material properties, and the boundary conditions are all consistent along the tangential direction [10]. The material properties of the main components defined by the corresponding B-H curves are given in Table 2. Meanwhile, the material of the shafts is non-magnetic stainless steel so that the simulation of the shafts can be negligible. The input current in the excitation coil was applied on the coil area as a current density load and the conditions parallel to magnetic flux were set to the boundaries. If the input current of the excitation coil is 2 A, the magnetic flux distributions and the magnetic field intensities of the clutches are presented in Figure 5. As shown in Figure 5a,c, the magnetic flux is strictly confined within the clutches. It can also be seen in Figure 5b,d that the magnetic field intensity of the working MRFs is larger than that of the surrounding area. For the disc-type MRC in Figure 5b, the magnetic field intensity of MRFs in two upper and lower MRF gaps is much larger than that in the side MRF gap. However, for the cylinder-type MRC in Figure 5d, the magnetic field intensity of MRFs in two upper and lower MRF gaps is much smaller than that in the side MRF gap.

**Figure 6:**
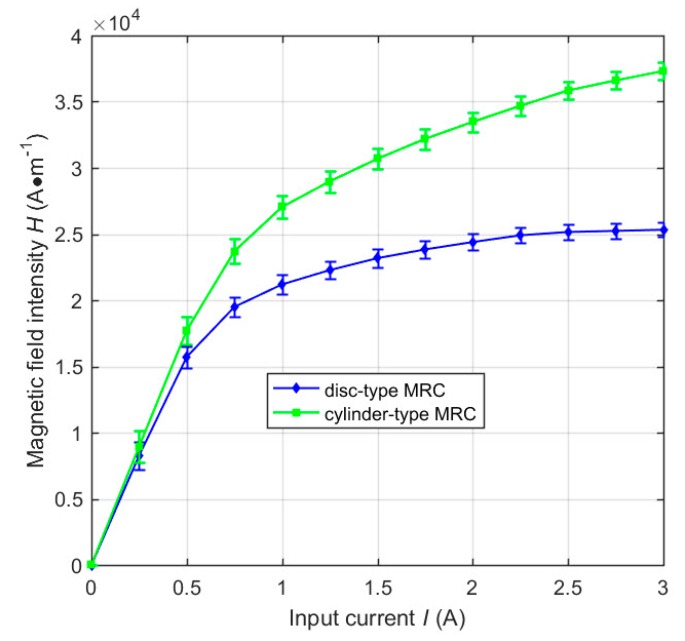
Relationship between the average magnetic field intensity of the working MRFs and the input current.

Besides, through ANSYS-Maxwell Post-processing, we took points every 1 mm along the working MRF gaps in Figure 5b,d, and thereby obtained the magnetic field intensity every 1 mm along the working MRFs. To simplify the calculation, we took averages among the values of the magnetic field intensity, and thus the average magnetic field intensity of the working MRFs of the clutches at *I* = 2 A were respectively obtained. The effect of the simplification equals that of the fact because of the constant total amount of the slip differential heat. Following this, we then calculated the magnetic field intensity distributions of the clutches similar as Figure 5b,d at different input currents by ANSYS-Maxwell. Proceeding as mentioned previously, the average magnetic field intensities of the working MRFs of the clutches at different input currents are computed as shown in Figure 6, which plots the relationship between the average magnetic field intensity of the working MRFs of the clutches and the input current. It can be noted that the average magnetic field intensity increases as the input current ranges from 0 to 3 A. The increasing speed continually decreases until it approximately keeps constant because of the magnetization saturation of the particles.

### 3.3. Thermal Field Analysis

Noted that there are two main heat sources within the clutches: (1) the slip differential heat of MRFs and (2) the Joule heating of the excitation coil due to the electrical current flow. An earlier study [30] showed that the former is a much more significant heat source, so the slip differential heat of MRFs was only considered in the present study. Moreover, there are two cases of the slip differential heat of MRFs: (1) In the absence of a magnetic field, the temperature rise of MRFs is caused by the liquid-to-liquid and particle-to-liquid friction, and (2) in the presence of a magnetic field, the temperature rise of MRFs is caused by the liquid-to-liquid, particle-to-liquid, and particle-to-particle friction. The experiment results [11] indicate that the former is much smaller than the latter so that the liquid-to-liquid and particle-to-liquid friction can be negligible. It is assumed that the slip power loss is all translated into slip differential heat for the temperature rise of MRFs [12]. Thus, the heating rate of the working MRFs is given by

(17) $$\phi_{m}=\frac{P_{m}}{V_{m}}$$

where *V_m_* is the volume of the working MRFs.

Alternatively, the relative velocity difference between the upper and lower walls *υ_τ_* and the area of the working MRFs *S* in the disc-type MRC and the cylinder-type MRC are respectively derived as

(18) $$v_{d}=\pi n\left(r_{2}+r_{1}\right)\;\mathrm{and}\;v_{c}=2\pi nr_{3}$$

(19) $$S_{d}=2\pi \left(r_{2}^{2}-r_{1}^{2}\right)\;\mathrm{and}\;S_{c}=2\pi r_{3}L$$

where *n* is the rotational speed difference of the clutches, *r*_2_ and *r*_1_ are respectively the inner and outer radii of the drive rotor of the disc-type MRC, *r*_3_ is the outer radius of the drive rotor of the cylinder-type MRC, and *L* is the length of the drive rotor of the cylinder-type MRC, see Table 1.

Substituting Equations (18) and (19) into (16) gives the slip power of the disc-type MRC and the cylinder-type MRC.

(20) $$P_{d}=\frac{\mu_{0}\mu_{k}\pi^{2}\chi_{0}^{2}M_{s}^{2}H{\left(I\right)}^{2}n\psi {\left(r_{2}+r_{1}\right)}^{2}\left(r_{2}-r_{1}\right)}{8{\left(\chi_{0}H+M_{s}\right)}^{2}}\left(7-\frac{3sin2\theta_{0}}{\theta_{0}}\right)$$

(21) $$P_{c}=\frac{\mu_{0}\mu_{k}\pi^{2}\chi_{0}^{2}M_{s}^{2}H{\left(I\right)}^{2}n\psi Lr_{3}^{2}}{4{\left(\chi_{0}H+M_{s}\right)}^{2}}\left(7-\frac{3sin2\theta_{0}}{\theta_{0}}\right)$$

It is known that the ferromagnetic particles are generally taken as pure iron particles where *μ*_0_*M_S_* ≈ 2.1 T so that M_S_ ≈ 1.671 × 10^6^ A·m^−1^ [20]. Given the rotational speed difference of the clutches, *n* = 200 rpm and other parameters are displayed in Table 3. When the input current *I* = 2 A and the average magnetic field intensity of the working MRFs of the disc-type MRC and the cylinder-type MRC are respectively 2.440 × 10^4^ A·m^−1^ and 3.348 × 10^4^ A·m^−1^. Therefore, the heating rate of the working MRFs of the disc-type MRC and the cylinder-type MRC are respectively *Φ_d_* = 2.036 × 10^7^ W·m^−3^ and *Φ_c_* = 2.651 × 10^7^ W·m^−3^ according to Equations (17), (20) and (21).

**Figure 7:**
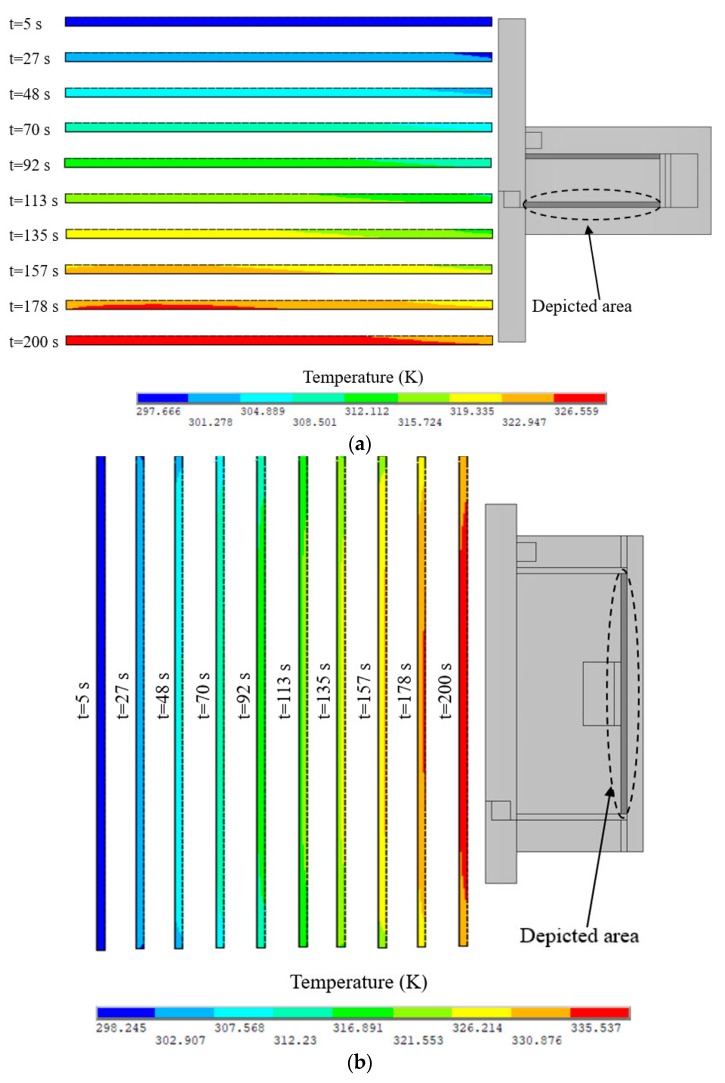
Temperature distributions of the working MRFs of the clutches under different slip times: (**a**) Disc-type MRC; (**b**) Cylinder-type MRC.

Similarly, the transient thermal simulations of the disc-type MRC and the cylinder-type MRC were carried out to calculate the temperature distribution and the temperature rise of MRFs by commercial finite element software ANSYS® 16.0 [31]. The PLANE55 element was used for the finite element modeling, and the material properties of components are the same as those in Table 2. The slip differential heat of MRFs in the disc-type MRC and the cylinder-type MRC were respectively applied on the working MRFs as the heating rate *Φ_d_* and *Φ_c_*. Moreover, we set some boundary conditions: The initial clutch temperature is 297.15 K, the outside air temperature is 297.15 K, all boundaries except the symmetrical boundary are convective heat transfer, and the convective heat transfer coefficient is 10 W·m^−2^·K^−1^. The temperature distributions of the working MRFs of the disc-type MRC and the cylinder-type MRC under different slip times are shown in Figure 7. It can be seen that the temperature of the working MRFs of the disc-type MRC and the cylinder-type MRC increases with the slip time, and the temperature distributions of the disc-type MRC and the cylinder-type MRC are relatively uniform.

## 4. Thermal Structure Field Analysis

It is significant to conduct the thermal structure field analysis to explore the effect of slip differential heat of MRFs on the structure since the structural sizes of MRFs, especially the gap thickness, are very small. It is known that MRFs demonstrate viscoelastic behavior in the presence of a magnetic field and, thereby, possess properties of general solid. However, in structural analysis, MRFs are a kind of mixture so that there are no available material property values, such as elastic modulus, Poisson’s ratio, and thermal expansion coefficient. A novel scheme for approximately determining them is presented in the present study. As shown in Equation (22), the shear stress τ of MRFs is related to shear strain *γ*.

(22) $$\tau =G^{*}\gamma$$

where G* is the complex shear modulus.

The complex shear modulus is written as [32]

(23) $$G^{*}=G^{\prime }\left(B\right)+iG^{\prime \prime }\left(B\right)$$

where *G*’(*B*) and *G*’’(*B*) are respectively the storage modulus and loss modulus of MRFs.

Where

(24) $$G^{\prime }\left(B\right)=3.11\times 10^{-7}B^{2}+3.56\times 10^{-4}B+5.78\times 10^{-1}$$

(25) $$G^{\prime \prime }\left(B\right)=3.47\times 10^{-9}B^{2}+3.85\times 10^{-6}B+6.31\times 10^{-3}$$

Thus, the tangential elastic modulus can be determined as

(26) $$E_{t}=2G^{*}\left(1+\mu \right)$$

where Poisson’s ratio *μ* is taken as 0.3.

The elastic modulus is related to the magnetic force under the same strain condition. According to Equation (6), the tangential and normal components of magnetic force are written as

(27) $$F_{mi}^{t}\left(\theta_{ij}\right)=F_{mi}\left(\theta_{ij}\right)\vec{t}$$

(28) $$F_{mi}^{n}\left(\theta_{ij}\right)=F_{mi}\left(\theta_{ij}\right)\vec{n}$$

where $\vec{t}$ and $\vec{n}$ are respectively perpendicular and parallel to the orientation of the magnetic field.

Thus, the normal elastic modulus *E_n_* is given by

(29) $$\frac{E_{n}}{E_{t}}=\frac{\int_{0}^{\theta_{0}}F_{mi}^{n}\left(\theta_{ij}\right)d\theta_{ij}}{\int_{0}^{\theta_{0}}F_{mi}^{t}\left(\theta_{ij}\right)d\theta_{ij}}$$

**Figure 8:**
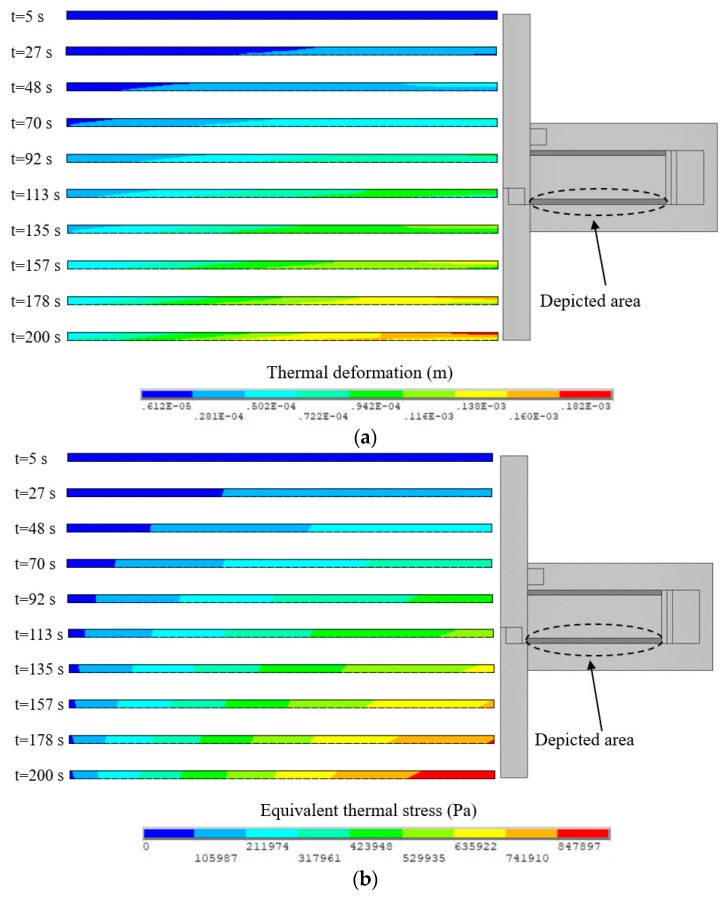
Results of the disc-type MRC: (**a**) Thermal deformation of MRFs; (**b**) Equivalent thermal stress of MRFs.

**Figure 9:**
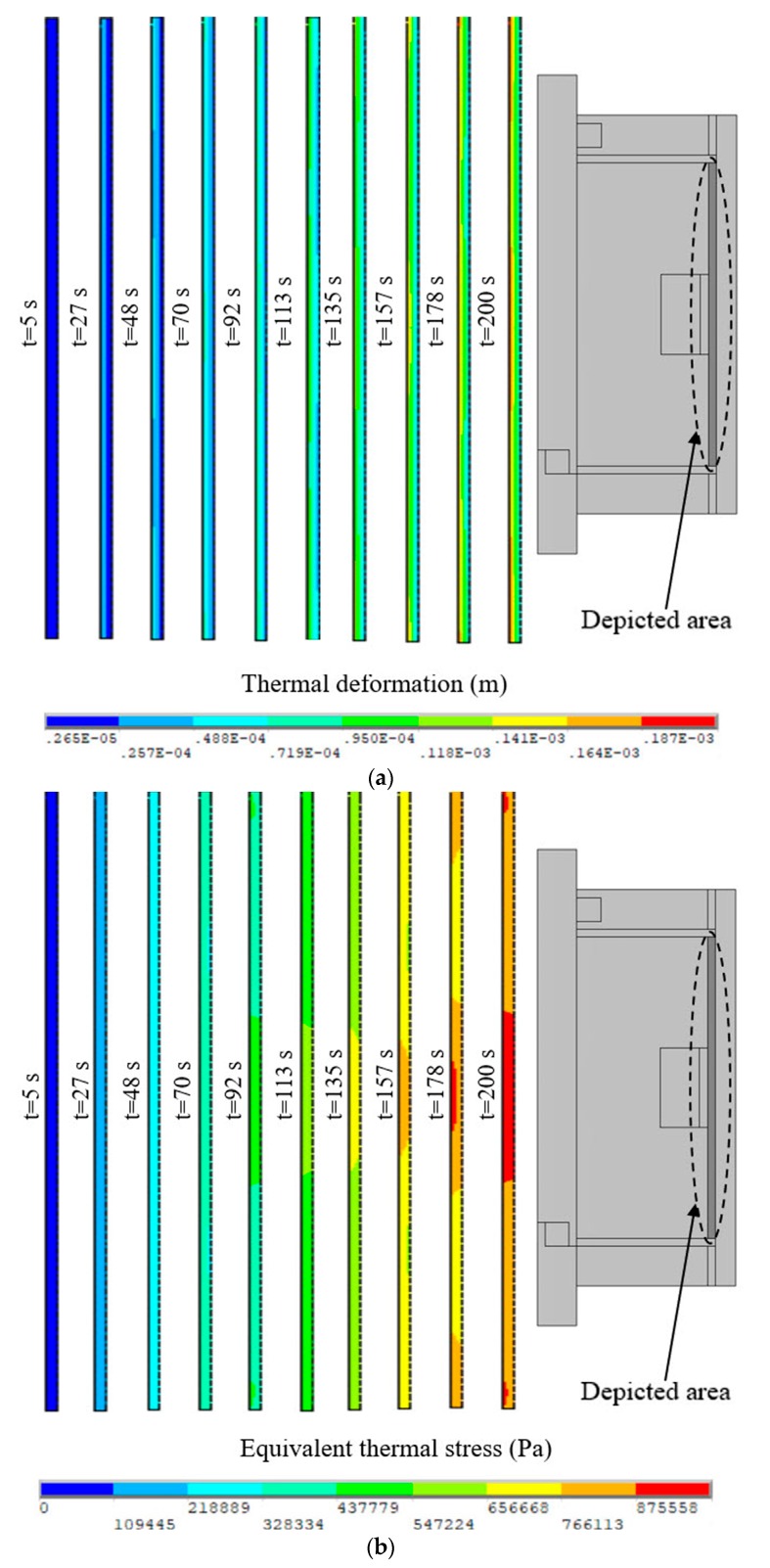
Results of the cylinder-type MRC: (**a**) Thermal deformation of MRFs; (**b**) Equivalent thermal stress of MRFs.

Computing Equation (29) gives *E_t_* = 5.296 × 10^6^ Pa and *E_n_* = 5.570 × 10^6^ Pa when the input current *I* = 2 A. Moreover, the thermal expansion coefficient of MRFs is replaced with that of the carrier liquid since the carrier liquid accounts for a large proportion of MRFs. The carrier liquid is generally silicone oil so that the thermal expansion coefficient is taken as 9.45 × 10^−5^ K^−1^. The thermal structure field simulations of the disc-type MRC and the cylinder-type MRC were also carried out to calculate the thermal deformation and equivalent thermal stress of MRFs by commercial finite element software ANSYS® 16.0 [31]. As presented in Figure 8, there is a gradient difference of thermal deformation and equivalent thermal stress between the left and right ends, and the difference gets more obvious with the increase of the slip time. However, as shown in Figure 9, there is a gradient difference of thermal deformation and equivalent thermal stress between the middle and both ends, and the difference also gets more obvious with the increase of slip time. These will have significant effects on the work performance of MRFs.

## 5. Results and Discussion

### 5.1. Temperature Variations of MRFs

**Figure 10:**
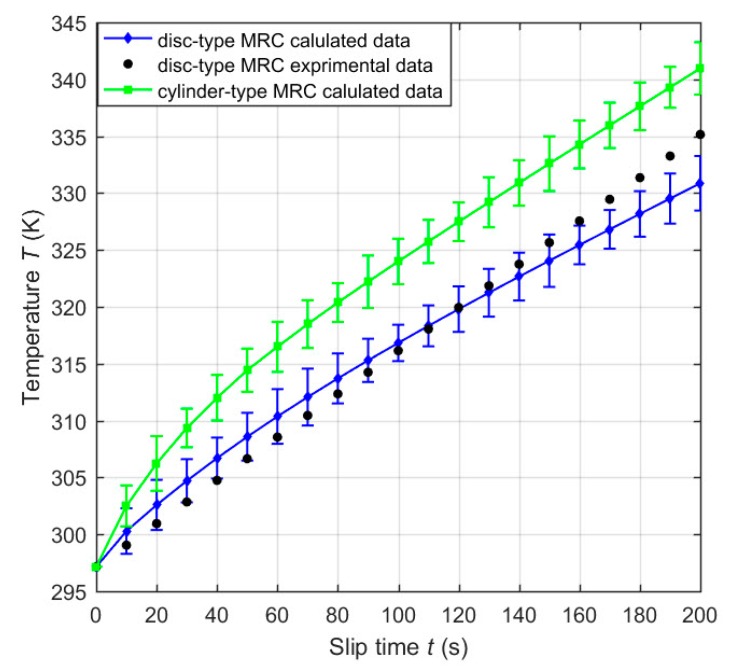
Temperature variations of the working MRFs of the clutches versus the slip time with the comparison of disc-type MRC experimental data [11].

It is known that mutual friction among the particles in each single chain will lead to a temperature rise of MRFs under the slip condition. Given the input current of the excitation coil *I* = 2 A and the rotational speed difference of the clutches *n* = 200 rpm, Figure 10 plots the temperature variations of the working MRFs of the clutches versus the slip time. It can be noted that the temperature of the working MRFs of the clutches increases with the slip time, which is due to the constant friction between the particles and the accumulated slip differential heat of the working MRFs within the clutches. Moreover, the temperature of the working MRFs of the disc-type MRC is slightly larger than that of the cylinder-type MRC. That is because the heating rate of MRFs of the disc-type MRC is smaller than that of the cylinder-type MRC under the same input current of the excitation coil and the same volume of the working MRFs. When the slip time ranges from 0 to 200 s, the temperature of the working MRFs of the disc-type MRC and the cylinder-type MRC respectively increases by 306.86 K and 316.96 K. Under almost identical conditions, the temperature rise of the working MRFs of the disc-type MRC is in reasonable agreement with the experimental result [11], and the minor difference can be partly attributed to the neglection of the heat of the carrier liquid.

### 5.2. Slip Power Variations of MRFs

**Figure 11:**
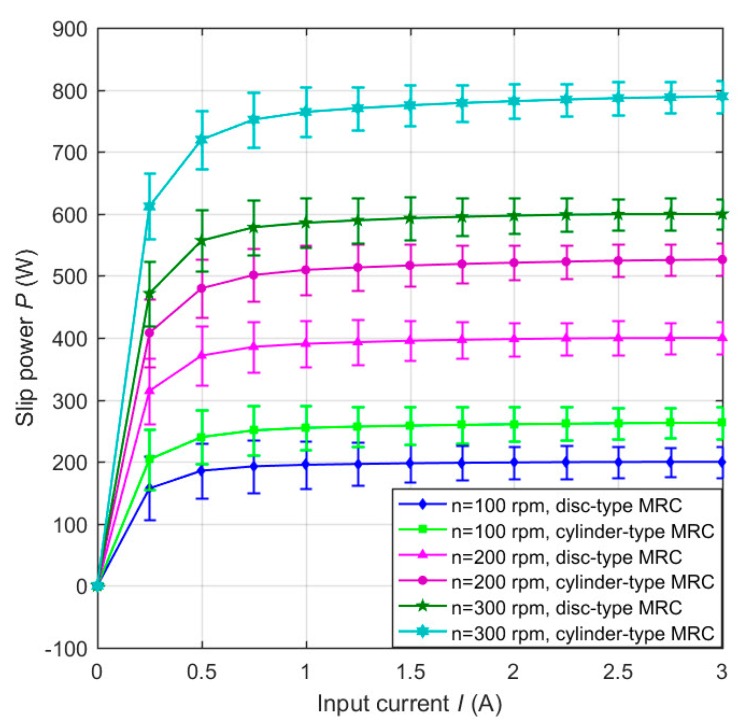
Effects of the input current and rotational speed difference on the slip power of the clutches.

Compared with the temperature affected by many external factors, the slip power can reflect the essence of slip differential heat of MRFs and mainly determine the temperature variation of MRFs. Wang et al. [12] studied the temperature variations of MRFs under different slip powers and demonstrated the temperature increases proportionally with the slip time. Figure 11 plots the slip power of MRFs of the clutches versus typical governing parameters, such as the input current of the excitation coil and the rotational speed difference of the clutches. It can be seen that the slip power of the working MRFs of the disc-type MRC and the cylinder-type MRC sharply increases at a small input current, which agrees with the tendency of the experimental measurements [14]. This condition is due to the strong magnetization of particles at a small magnetic field intensity. From there, the slip power then increases a little or even nearly keeps constant after *I* = 0.5 A, which also agrees with the tendency of the experimental measurements [9]. That is due to the fact that the magnetization saturation of particles are being approached. Under the same conditions, the slip power of the working MRFs of the disc-type MRC is slightly smaller than that of the cylinder-type MRC. That is because the average magnetic field intensity of the working MRFs of the disc-type MRC is slightly smaller than that of the cylinder-type MRC, see Figure 6. Moreover, the slip power of MRFs of the clutches increases proportionally as the rotational speed difference of the clutches increases, which agrees with the tendency of experimental measurements [11]. That is because, as the rotating speed increases, the rotational speed difference and the transmission torque all increase, and the slip power increases with the increase of the transmission torque.

### 5.3. Effect of Slip Differential Heat on the Structure of MRFs

**Figure 12:**
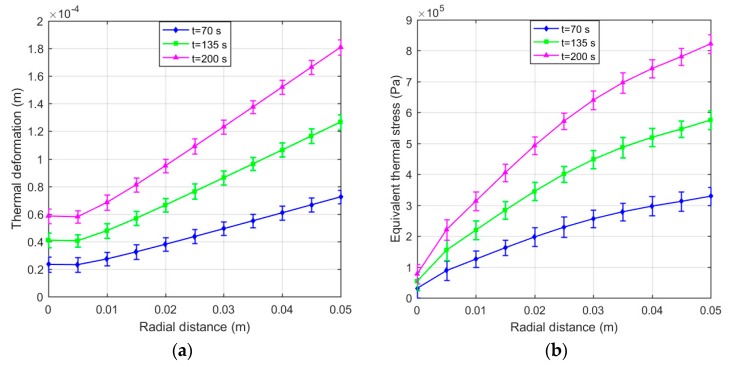
Thermal deformation and equivalent thermal stress of MRFs of the disc-type MRC along the radial working gap. (**a**) Thermal deformation; (**b**) Equivalent thermal stress.

**Figure 13:**
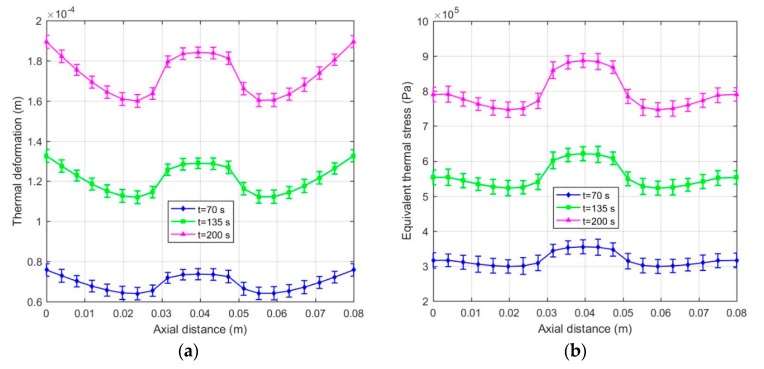
Thermal deformation and equivalent thermal stress of MRFs of the cylinder -type MRC along axial working gap: (**a**) Thermal deformation; (**b**) Equivalent thermal stress.

The temperature effect on the structure of MRFs is not negligible with the accumulation of slip differential heat under the slip condition. Figure 12 plots the thermal deformation and equivalent thermal stress of MRFs of the disc-type MRC along the radial working gap at different slip times. It can be noted that the thermal deformation and equivalent thermal stress increase with the slip time, and the variations of the thermal deformation and equivalent thermal stress are generally similar but differ in certain details. This phenomenon is due to the accumulation of slip differential heat with the slip time and the varying temperature distributions along the radial working gap. Meanwhile, Figure 13 plots the thermal deformation and equivalent thermal stress of MRFs of the cylinder -type MRC along the axial working gap at different slip times. It can be seen that the thermal deformation and equivalent thermal stress are corrugated and symmetrical along the radial working gap, and other disciplines are the same as that of the disc-type MRC. On the whole, the thermal deformation and equivalent thermal stress of MRFs of the disc-type MRC are slightly larger than that of the cylinder -type MRC, and the values that respectively reach 10^−4^ m and 10^5^ Pa are all within the scope of the license under the conditions of the present study. Since the micro-structure of MRFs are the chains consisting of the micron-sized particles, the thermal deformation and equivalent thermal stress of slip differential heat undoubtedly have certain influences on the micro-structure of MRFs.

## 6. Conclusions

In the present work, a novel theoretical model for determining the slip differential heat of magnetorheological fluids (MRFs) subjected to shear mode operation is presented. It consists of the micro-macro model of slip differential heat of MRFs and the application to the disc-type magnetorheological clutch (MRC) and cylinder-type MRC. We conduct a thermal structure analysis of MRFs comprising thermal deformation and equivalent thermal stress. The model takes into account the effect of each of the main influencing factors, such as the input current of the excitation coil, the rotational speed difference of the clutches, the size and the volume fraction of particles, the saturation magnetization of particles, and the structural size of the clutches, etc., on the slip differential heat of MRFs. Through the prediction of the temperature variations of MRFs under different slip times, the effectiveness of the proposed model is validated and compared with the experimental measurements of Reference [11]. Moreover, the effect of typical governing parameters on the slip power of MRFs and the influence of slip differential heat on the structure of MRFs are investigated individually. The main conclusions are obtained as follows:

1. The temperature of MRFs increases with the slip time due to the accumulation of slip differential heat.
2. The slip power increases proportionally with the increase of the rotational speed difference of the clutches, but it first increases dramatically and then increases a little or even keeps constant at the input current *I* = 0.5 A with the increase of the input current.
3. The thermal deformation and equivalent thermal stress of MRFs are uneven along the working gaps, and their values that respectively reach 10^−4^ m and 10^5^ Pa are all within the scope of the license under the conditions of the study. However, this undoubtedly has a certain influence on the micro-structure of MRFs. An increase in micro-structure detail can indicate the effect of the thermal deformation and equivalent thermal stress of MRFs on the worn surfaces of the friction disc and cylinder, if desired [16].
4. All the above results of the disc-type MRC are slightly smaller than that of the cylinder-type MRC under the same conditions including the same input current of the excitation coil, the same rotational speed difference of the clutches, and the same volume of MRFs.

## Figures and Tables

**Table 1:** Design parameters of the clutches.

Parameter	Value
Shaft radius	0.01 m
Drive rotor radius (disc-type MRC)	0.06 m
Drive rotor radius (cylinder-type MRC)	0.044 m
Drive rotor length (disc-type MRC)	0.02 m
Drive rotor length (cylinder-type MRC)	0.08 m
MRF gap thickness	0.001 m
Number of coil turns	200

**Table 2:** Materials for main components of the clutches.

Component	Material
Drive shaft	Stainless steel (AISI 304)
Driven shaft	Stainless steel (AISI 304)
Drive rotor	Fine carbon steel (AISI 1020)
Driven rotor	Fine carbon steel (AISI 1020)
Working gap	MRFs (SG-MRF2035)
Isolation ring	Brass (UNS C27400)
Excitation coil	Copper

**Table 3:** Calculation parameters.

Parameter	Value
Permeability in free space *μ*_0_	4π × 10^−7^ A·m^−1^
Friction coefficient *μ_k_*	0.15
Initial susceptibility *χ*_0_	1000
Saturation intensity of magnetization *M_S_*	1.671 × 10^6^ A·m^−1^
Volume fraction of particles *ψ*	20%
Maximum tilt angle *θ*_0_	π/4 rad
Particle radius *R*	5 × 10^−6^ m
